# Effect of modeling porous media on the response of gamma-gamma well-logging tool

**DOI:** 10.1038/s41598-020-63323-x

**Published:** 2020-04-22

**Authors:** Fatemeh S. Rasouli, S. Farhad Masoudi

**Affiliations:** Department of Physics, K.N. Toosi University of Technology, P.O. Box 15875-4416, Tehran, Iran

**Keywords:** Physics, Applied physics

## Abstract

The well logging is known as a technique of making petrophysical measurements in the sub-surface earth formations through a drilled borehole to reach the characterization of the physical properties of rocks and fluids. Considering the fact that reservoirs are complex fractured media which the fluid can flow through the porosities, the distribution model of oil in the medium needs to be investigated in detail and to be well quantified. To study this effect, a typical gamma-gamma logging tool containing ^137^Cs source and two NaI detectors was modeled by using the MCNPX code. The medium was filled with numerous matrix-shaped blocks, each including rectangular cubes for modeling the oil flow in the formation. For an arbitrary set of oil concentrations and various formation materials, the response of the detectors for this model was studied. Taking into account the results corresponding to the traditional homogeneous mixture model for the formation, it was found that the deviations between the count rates for two models reach to about 10% and 22% for short spacing and far spacing detectors, respectively. The results also show that the slopes of the straight-line fits to the count rates, which is important for the evaluation of the density, deviate between about 73.3% and 53.8% for two simulated models. Investigating the effect of the presence of the drilling fluid on the count rate of the proposed model showed that for a given thickness of mudcake and the formation density, both detectors show approximately the same percentage of change in counting rate. However, these counts for the proposed model deviate from those of the mixture model between 5.1% and 28%. It can be concluded that defining a model for describing heterogeneities of a natural porous medium can effectively account for the prediction of density measurement in logging tools.

## Introduction

As one of the most prominent applications of radiation detection, the well logging technique is widely used for density measurement. The necessity of the petroleum industry to the estimation of hydrocarbon accumulations was the biggest motivation for the growth of well logging. This method which is alternate to the analysis of cores, side-wall samples, and cuttings can be categorized into three broad disciplines: electrical devices, nuclear measurements, and acoustic measurements. A well logging tool, sometimes referred to as sonde, consists of a cylindrical cover containing a set of facilities^1^ and are designed to respond to the content of earth formations. The other side of well logging is data analysis techniques which have been progressed significantly over the past few decades. Excellent discussions covering them can be found elsewhere^2,3^.

Nuclear measurements which the present study has focused on, is based on employing gamma rays and neutrons for investigation of the properties of both the formation and the contained fluids. A typical nuclear logging tool consists of a neutron or gamma source and one or more detectors for recording the scattered and secondary radiations generated due to the interaction of the source particles with the formation. The number of these detected particles are used to infer characteristics of the surrounding rock. Nuclear well logging tools can be also designed without a radioactive source to respond to the natural gamma radiation arisen from the few isotopes in the formation^4,5^. By employing either of these methods, if Compton scattering is the main interaction, the transmission of gamma rays through matter can be interpreted as the electron density which directly relates to the density of the medium. The technology of density measurement by using Compton scattering of gamma rays in the material has been introduced by the pioneering work of Zak and Smith for hydrocarbon exploration in the petroleum industry^6^.

It is well known that the formation density is directly proportional to the formation porosity, a quantity which is defined as the fraction of the volume of voids over the total volume^7,8^. It is therefore a dimensionless quantity and is an important factor in the petrophysical studies on resistivity measurements considering the fluid saturation. A porous material is usually defined as a solid containing voids or holes that are either connected or non-connected. These void spaces may be filled with a liquid, e.g. water or oil. Reservoirs are known as natural porous media; As the rocks were being formed during the geological period, these storage space generated under *in-situ* stress or chemical action during the long geological period. Well logging affords to give measurements of the formation characteristics as well as the fluids present in its pore spaces.

Typical gamma-gamma logging devices consist of a gamma source and incorporate two or more analogous detectors (generally NaI) in a housing that shields them from direct radiations of the source. The tool is suspended on a cable or wire and can be run in the borehole at the end of drilling operations. The detectors measure the returning Compton-scattered gamma rays to reflect the shale content and to determine the electron density. The logging tool is to be manufactured after appropriate design for configurations of the tool. The designing processes generally benefit from the Monte Carlo method, known as an appropriate way for transport of particles through a chosen target and handling calculations in complex three-dimensional geometries. There are a number of worthy published works discussing on investigation of several factors governing the performance of the logging tools and investigation of an optimized logging tool using the Monte Carlo method. These studies include designing the borehole geometry, setting the optimized number of detectors and their position in the device, prescribing source position and the effect of the angle of the emitted photons on the efficiency of the tool, as well as finding mathematical derivations, formulas, and methods for prediction of the formation density^9–14^.

In the petroleum industry, well logging plays a fundamental role to interpret downhole conditions and ideally is expected to provide measurements of the formation compositions and the fluids in the pore spaces. Owing to that the realistic formation is a fractured medium containing the fluid flows in its pores, the proper description of a porous medium or fractured rock can be of high importance in developing the design of gamma-gamma well logs. In other words, a realistic medium in which the well logging experiments are performed is a heterogeneous composition of pores, providing penetration of fluids in the medium, and the formation materials. As delivering accurate and reliable measurements is obviously a challenge, simulation studies try to present a description of the oil reservoir as precisely as possible. However, it is unfortunate that the role of the simulation of complex fractured media is overlooked in the literature discussing the effects of parameters on the response of density measurement tools^9–14^, and it is routinely assumed that the formation is a homogeneous media with various weight fractions of oil.

Considering the mentioned points, the present work has been devoted to the modeling of fluid flow in fractured reservoirs and investigation of the necessity of considering the formation as an inhomogeneous fractured medium for gamma-gamma logging tools. Owing that the response of the detectors located in the logging tool is the key quantity for estimation of the density of the medium, it has been considered as a criterion to assess the effect of detailed modeling for the distribution of pores in the formation on simulation studies. The results clarify that weather the corrections corresponding to the precise description of the medium are needed in logging tool designs. The study is structured as follows: A typical gamma-gamma logging tool located in a medium with the central borehole is introduced. An inhomogeneous model of formation containing various percentages of porosity with random distribution in the medium is proposed. The pores are supposed to be saturated with oil. For a given formation material, the model is used for studying the behavior of the detector’s response against the oil concentrations. By comparing the obtained results with those of the traditional model with a homogeneous mixture of formation and oil, the effect of simulation of porosities in the radiation-based well logging technique is discussed. Furthermore, by considering formation materials with various densities, the effect of using the proposed model on density evaluation is inquired. The study is extended to estimate the effect of the presence of drilling fluids on the gamma-ray counts.

## Materials and Methods

Monte Carlo is known as an appropriate method for the transport of particles through a chosen target. Though it is a non-deterministic method and the accuracy is limited by repeated random sampling, it is the only way for accurate and reliable prediction of the behavior of the particles in the complicated media. The uncertainty of Monte Carlo’s results depends on the number of histories. Accordingly, an adequate number of particles should be tracked to achieve a given accuracy and reliable results. In the present work, simulations and particle tracking in the medium are carried out using the well-known and tested Monte Carlo N-Particle eXtended code (MCNPX). The details of the simulations and models we used are as presented in the following subsections.

### Logging tool

The simulated logging tool consists of a typical 50 mCi ^137^Cs gamma source. The energy spectrum of the emitted photons are below the threshold for pair production (maximum 661.6 keV). This isotope has a half-life of 30.17 years and therefore provides a stable intensity during a reasonable period of time. Two analogous NaI detectors have been simulated at the different fixed distances from the source position along the tool surface. They are generally named as near and far detectors. In our simulations, the near and far detectors have been located at the distances of 20 cm and 31 cm from the source position, respectively. The energy range of detected gamma rays was set as 200 to 600 keV. The source is shielded from the two detectors so that only scattered gamma radiation is detected.

**Figure 1:**
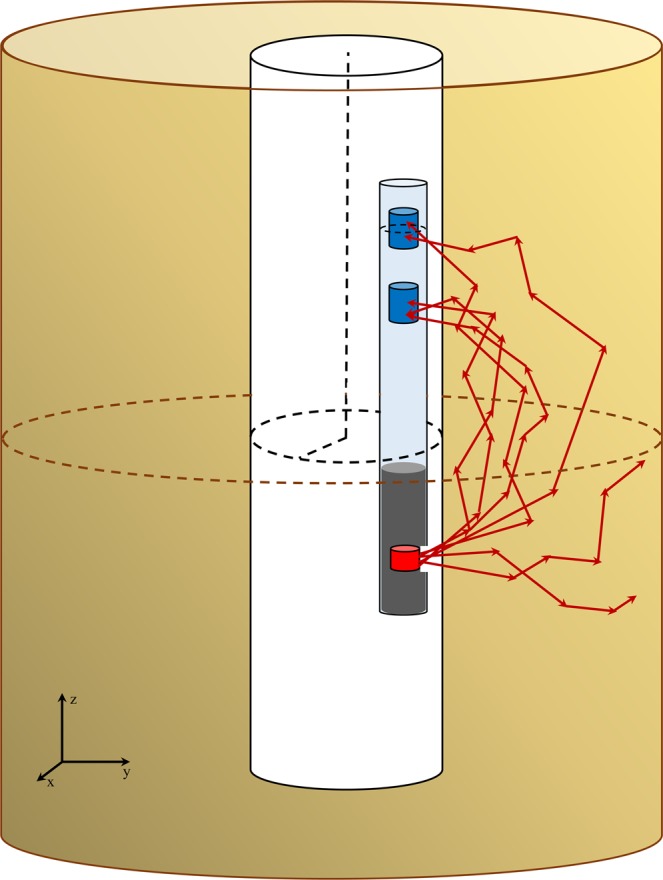
A schematic view of the simulated logging device in the borehole. The device includes a ^137^Cs gamma source and two NaI detectors. The Pb shield is placed between the source and the near detector to avoid detecting the directly arrived gamma rays. The formation encircles the borehole. This graph, as well as those in Figs. 2 and 3, have been drawn by LibreOffice 6.3.4, https://www.libreoffice.org/download/download/?type=deb-x86_64amp;version=6.3.4amp;lang=en-US.

A schematic view of the simulated device in the borehole is shown in Fig. 1. The tool is connected to an electrical cable, not shown in the figure, to lower the tool into the well. As the figure shows, the formation has been considered as a cylinder surrounding a cylindrical borehole of 12 cm in diameter. The height of the formation is 100 cm and its diameter is determined considering the effective depth for gamma rays in the medium.

### Formation material

Interaction of gamma rays through Compton scattering depends on the density of the scattered electrons which is directly proportional to the density of the formation. As is well-known, the reduction of the source strength *N*_0_ after passing a thickness of material *x* is given by:

1 $$N={N}_{0}{{\rm{e}}}^{-\rho \frac{Z}{A}{N}_{A}\sigma x}$$

where $\rho \frac{Z}{A}{N}_{A}$ shows the density of electrons in material of mass density *ρ*, and *σ* is the cross-section for Compton scattering. It is therefore obvious that the response of the detector ideally corresponds to the slope of the logarithm of the counting rate as a function of density. It is a motivation to use the recorded gamma rays in the detectors to estimate the formation density.

In the present study, seven different minerals have been considered as the formation material. These materials, listed in Table 1, have chosen so that they cover various mass densities compared with that of the oil which is supposed to flow through the formation. The ratio of these densities to that of oil ranges between 1.96 to 3.7.

**Table 1:** List of the minerals used as the formation in this study. The chemical formula and the mass densities are also reported. The data have been taken from ref. ^15^.

Formation	Chemical formula	*ρ*(g.cm^−3^)
Carnallite	KCl.MgCl_2_.6H_2_ O	1.61
Sylvite	KCl	1.984
Gypsum	CaSO_4_.2H_2_ O	2.32
Quartz	SiO_2_	2.648
Calcite	CaCO_3_	2.71
Dolomite	CaCO_3_.MgCO_3_	2.87
Magnesite	MgCO_3_	3.037

During the drilling of boreholes into the earth, the drilling fluid, generally known as mudcake, is used for the technical reasons to facilitate the drilling process such as carrying out drill cuttings and keeping the drill bit cool and clean during drilling. It can be expected that when a material is placed between the logging tool and the formation, there will be a perturbation in counting rate and the density estimated using the detectors will no longer be equal to the formation density. With the correction term Δ*ρ* which is traditionally added to the far detector density (*ρ*_*fd*_), the density can be estimated as^15^:

2 $$\rho ={\rho }_{fd}+\Delta \rho $$

The final section of the present study seeks to estimate the effect of the presence of mudcake in a simulated medium. There are many types of drilling fluids including water-based and oil-based muds, and gaseous drilling fluids^16^. Barite (BaSO_4_) is the commonly used weighting agent that is employed to increase the density of a drilling fluid. In this work, this material is used as the weighting agent for a water-based mudcake with a typical thickness of 1 cm. The penetration of the mudcake into the formation is neglected.

### The porous medium

The natural media in many fields of science can be considered as disordered porous media, and the fractures are present in soil, wood, concrete, glaciers, ceramic, etc. The subsurface rocks, which are of high importance in both hydrology and petroleum engineering, are also examples of such media. The rock texture consists of grains of various shapes and sizes and its pore structure is extremely complex. The existence of fractures, ranging from millimeters to centimeters, can provide fluid movements in the rocks and therefore the porosity is a measure of space available for the storage of hydrocarbons. As a result, studying the behavior of fractures in oil reservoirs and the physics governing fluid flow in these media is one of the main topics of research. While the naturally fractured media are very complicated, they are generally characterized by simplified shapes, including the matrix models^7,17–19^.

**Figure 2:**
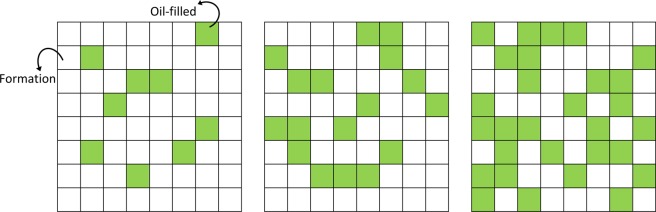
Examples of 8 × 8 matrix-shaped blocks designed for oil flow in the pores of the formation. The oil-filled sites are shown in color. In these models, quartz has been considered as the formation material and the matrix blocks from left to right belong to the oil concentrations (weight fraction) of 5, 10, and 20%, respectively.

**Figure 3:**
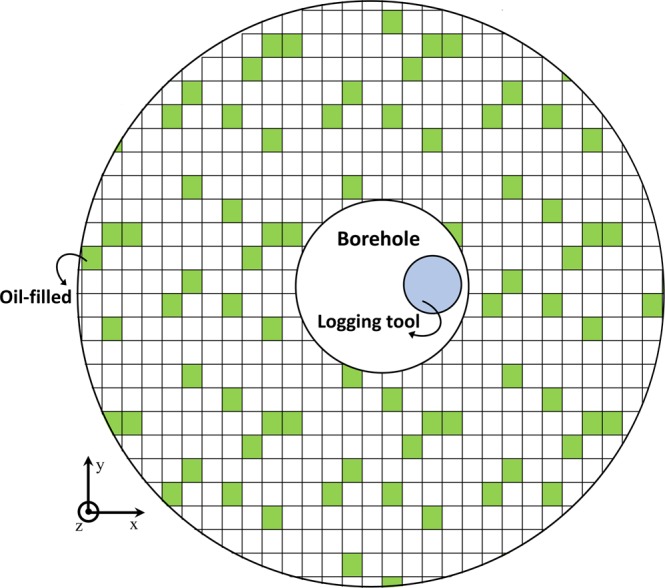
A schematic top cross-sectional view of the medium filled with matrix blocks. The oil-filled sites are shown in color. The central borehole and the logging device are also shown. The number of rectangular cubes and dimensions of the geometries are not to scale.

The present study essentially seeks to investigate the effect of simulating a porous media rather than a homogeneous mixture material on the response of the detector. To address this concern, the formation is filled with copious 8 × 8 matrix-shaped blocks. This matrix includes rectangular cubes of 0.1 × 0.1 × 100 cm^3^ for modeling of fluid (oil) flow in the formation. These rectangular cubes are loaded with either formation material or oil. The number of oil-filled sites is changed depending on the concentration of oil in the formation. Taking into account the fact that the most natural and artificially porous media have a random structure which can be only described in the statistical term, the oil-filled sites are distributed randomly in the matrix blocks. Strictly speaking, it is assumed that all the arbitrary distributed pores in the formation are saturated with oil. Accordingly, the term *mass concentration* refers to porosity. Figure 2 shows examples of designed matrix blocks for three oil concentrations where quartz has been used as formation material. Strictly speaking, this model is a heterogeneous arrangement of rectangular cubes which are randomly filled with either oil or formation material. A schematic top cross-sectional view of the medium filled with matrix blocks with the central borehole is presented in Fig. 3. Hereupon such a heterogeneous medium is named *porous model*.

For the model named *mixture*, the medium is filled with the homogeneous mixture of formation material and different concentrations of oil, considering the number of given oil-filled sites in the matrix blocks of the porous model. This model is used to test the enhancement/decrement of the count rate of the gamma rays in the detectors for the generally used approach for simulation of the medium compared with those of the heterogeneous porous media.

In both models, the oil concentration (weight fraction) is set in the range between 5 to 30%. In the coming sections, the detector response for the porous model is denoted by *N*_*p*_ and the corresponding value for the homogeneous mixture model is described by *N*_*m*_.

## Results

**Figure 4:**
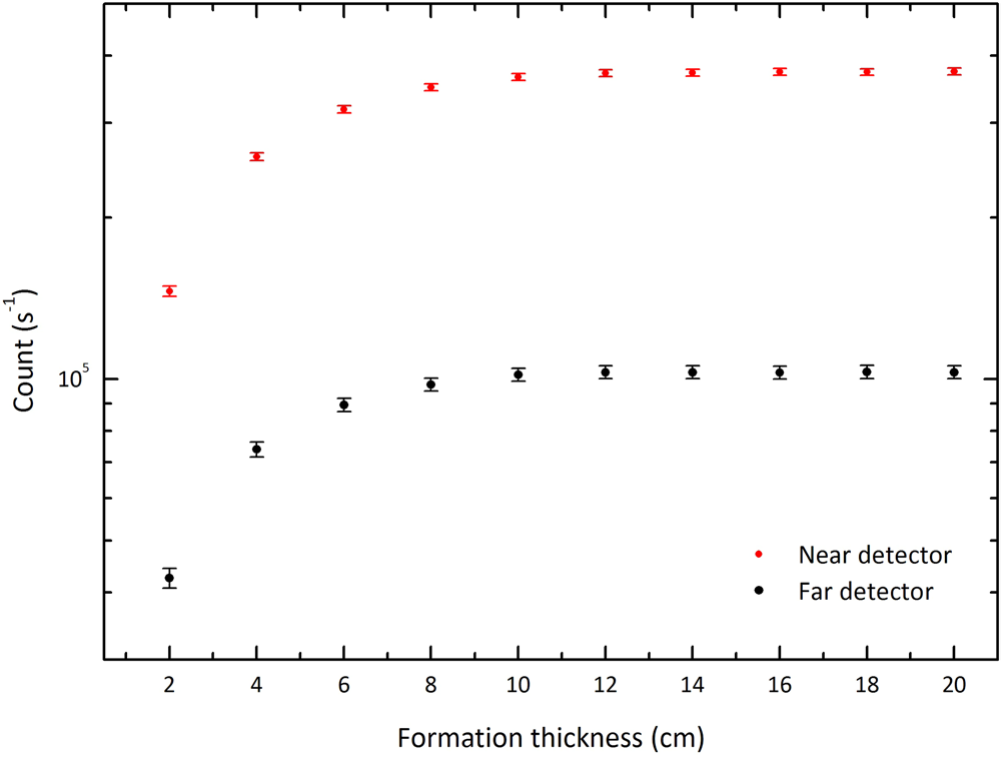
The response of the near and far detectors for various thicknesses of quartz as a typical formation material. Error bars indicate the relative uncertainties. This plot, as well as those in Fig. 5. to 10, have been drawn by QtiPlot 0.9.9.14, https://www.qtiplot.com/.

To save the computation time of the Monte Carlo method, the first step has been devoted to finding the optimized thickness of the enclosure to be used as an infinite medium where the calculations have to take place. Figure 4 shows the detected gamma rays in two NaI detectors for various thicknesses of the medium where quartz has been used as the formation material. As discussed previously, these data determine the effective depth of gamma rays in the formation. As the results show, the number of detected particles in the both detectors remains constant by about the depth of 10 cm. Considering these results, the thickness of the formation has been set to 12 cm. Taking into account the radius of the central borehole, the outer radius of the medium is 18 cm.

**Figure 5:**
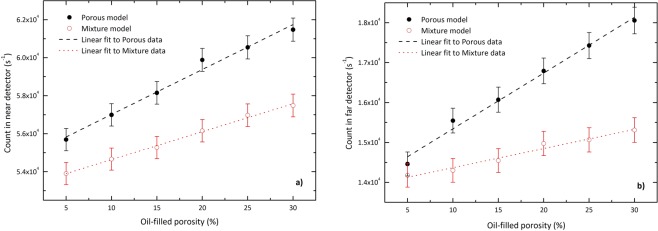
The response of (**a**) near detector and (**b**) far detector for different oil concentrations corresponding to the porous model and the homogeneous mixture model where quartz is the formation material. Error bars indicate the relative uncertainties and the straight-lines are linear fits to the data.

Employing the matrix blocks designed, the medium containing various oil concentrations have been modeled. Figure 5 shows the count rate of gamma rays in the detectors as a function of oil concentration where quartz is the formation material. The figures also compare these values with those of the homogeneous mixture model. As the figures suggest, for both the models, the count rate increases with the increment of the porosity linearly. The results also show the distinct difference between the responses of the detectors corresponding to two models. The symbol defined as *δ*(*N*_*p*_) = (*N*_*p*_/*N*_*m*_) − 1 is introduced to show these deviations. For the oil concentrations investigated in this work, *δ*(*N*_*p*_) for the near detector ranges between 3.3% and 7%. For the far detector, this value ranges between 2% and 18%.

Though the count of gamma rays in two models behaves linearly with oil concentration, the slopes of these straight-line fits are not the same. For the oil concentrations tested in this work, the slope of the linear fit corresponding to the porous model deviates from that of the homogeneous mixture model by about 60.7% and 191% for near and far detectors, respectively. By way of explanation, the variation of the count rate vs the oil concentration in the porous model is faster than that of the homogeneous mixture model.

**Figure 6:**
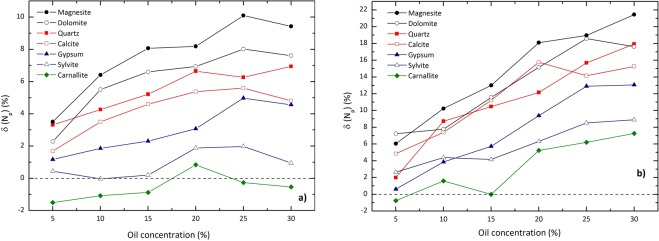
Deviations (in percent) from *N*_*m*_ of *N*_*p*_ for (**a**) near detector and (**b**) far detector for different oil concentrations. The curves correspond to the formation materials listed in Table 1. The lines are guides to the eye.

Similar calculations have been carried out for other formation materials tabulated in Table 1. To examine the difference between the performance of the models suggested, deviations from detectors’ responses correspond to the porous model of those to the homogeneous mixture model for different oil concentrations have been calculated. The results have been presented in Fig. 6. As the curves show, *δ*(*N*_*p*_) ranges between about −2 to 10% for the near detector, and between about −1 to 22% for the far detector.

**Figure 7:**
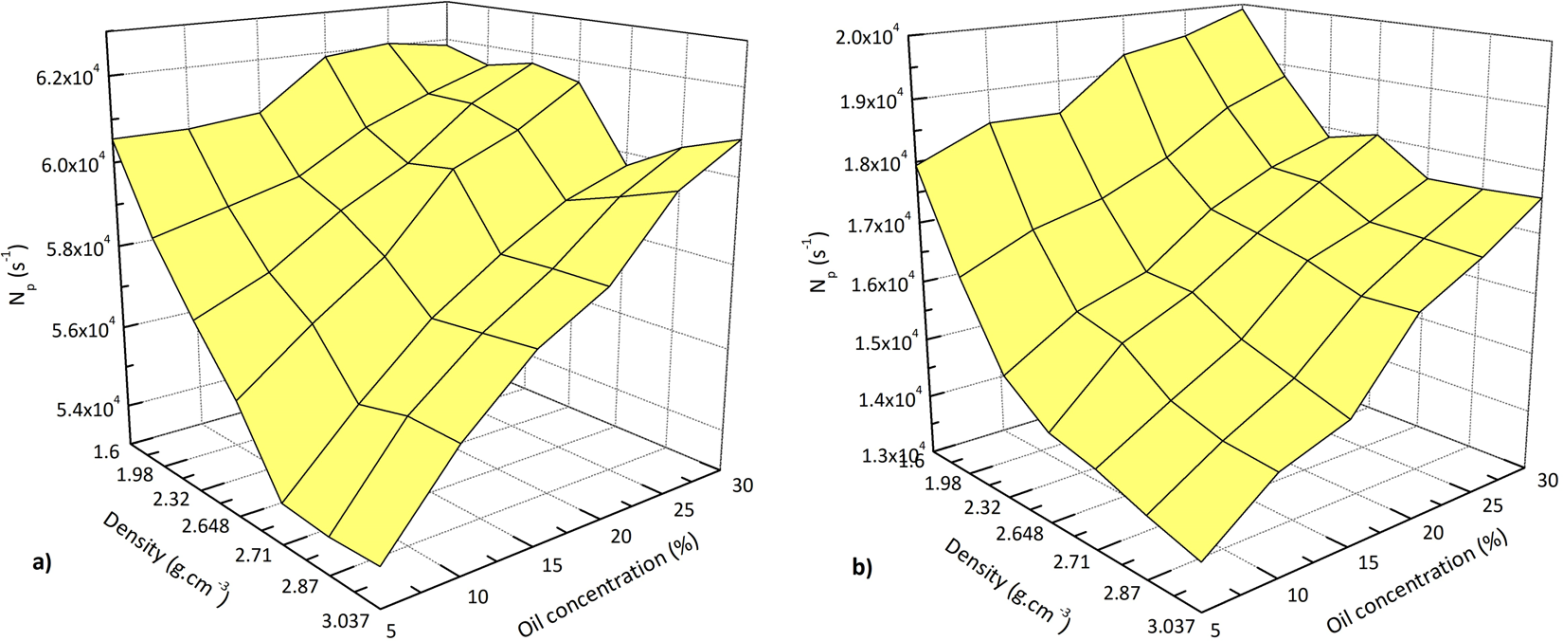
A 3-D plot of dependency of the count rate on both oil concentration and the formation material (density) for (**a**) near detector and (**b**) far detector in the porous model.

Figure 7 reports a 3-D plot of dependency of *N*_*p*_ on both oil concentration and the formation material (assigned as the formation density) for the porous model. For all densities tested, the minimum and the maximum values of *N*_*p*_ belong to the smallest and largest oil concentration considered in this work, respectively. As is expected, for the least value of oil concentration, the response of the detectors for the formations with lower densities is more than those of higher densities. As an example, for oil concentration of 5%, *N*_*p*_ for carnallite deviates from that of magnesite by about 12.3% and 30.5% for near and far detectors, respectively. However, owing to that the formation materials with higher densities are more sensitive to the variations of oil concentrations, for the concentration of 30%, these deviations decrease to about 1.6% and 13.4%.

**Figure 8:**
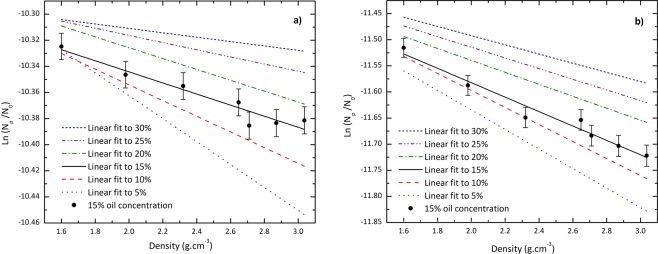
Counting rate response of (**a**) near detector and (**b**) far detector for variation of formation density. For the purpose of brevity and to prevent tangling, only the data corresponding to arbitrary oil concentration of 15% are shown. For other concentrations, the linear fits are reported.

Equation (1) shows an exponential relationship between the counting rate and the formation density. For a given concentration, the response of the detectors can be used for estimation of the formation density and consequently identifying its material. The results of Fig. 7 suggest that the counts in the porous model needs modifications to be used for density evaluation. To address this issue, the counting rates of the detectors for variation of formation density have been investigated. The results are presented in Fig. 8. For the sake of brevity and to prevent tangling, this figure shows just the data corresponding to the arbitrary oil concentration of 15%. For other concentrations, the linear fits to the relative data have been shown.

**Figure 9:**
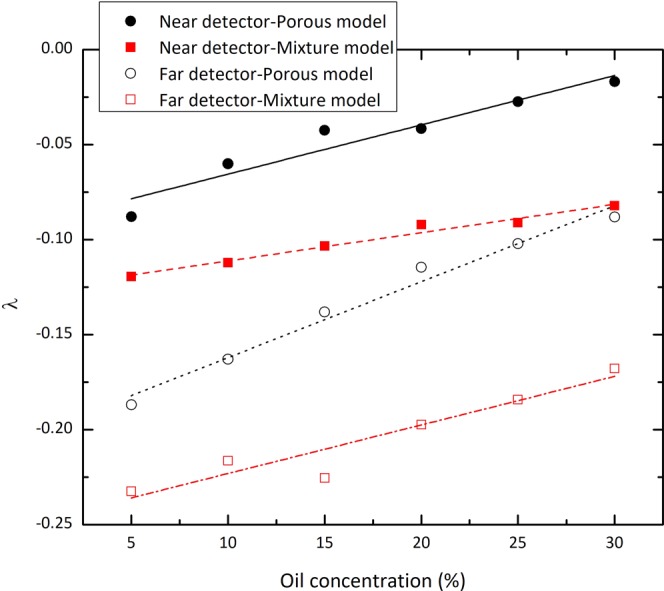
The values of *λ* versus the oil concentration of the medium for near and far detectors in porous and homogeneous mixture models. The straight-line fits are also shown.

As one can see from Fig. 9, the slopes of these straight lines, denoted by *λ*, are concentration-dependent which behave linearly with oil concentration as Eqs. (3) and (4) for near and far detectors, respectively:

3 $${\lambda }_{p}=0.0026\Phi -0.090$$

4 $${\lambda }_{p}=0.004\Phi -0.2$$

where the symbol Φ is used to show the oil concentration and the subscript *p* is indicative for the porous model. According to the results, the shorter spacing detector has less density resolution compared to the farther detector. Furthermore, the curves imply that the slope of the linear fit corresponding to Φ = 5% deviates from that of 30% by about 422.7% and 112.2% for near and far detectors, respectively. However, the intercepts are less sensitive to the oil concentrations so that this quantity for the linear fit corresponding to Φ = 5% deviates from that of 30% by about −0.88% and −0.49% for shorter and farther spacing detectors, respectively.

Figure 9 also includes the behavior of the slopes of straight line fits for the homogeneous mixture model. These values can be described in Eq. (5) for near detector and in Eq. (6) for far detector:

5 $${\lambda }_{m}=0.0015\Phi -0.12$$

6 $${\lambda }_{m}=0.0026\Phi -0.25$$

where subscript *m* is representative of the mixture model.

**Figure 10:**
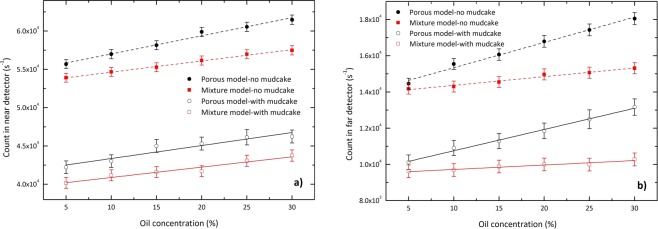
The values of *N*_*p*_ and *N*_*m*_ in (**a**) near detector, and (**b**) far detector for various oil concentrations in the presence and the absence of drilling fluid where quartz has been considered as the formation material. The straight-line fits are also shown.

To assess the effect of using drilling fluid on the response of the detectors for two discussed models, a typical 1 cm thickness of mudcake has been added to the borehole wall. Figure 10 shows *N*_*p*_ in near and far detectors for a set of oil concentrations in the presence and the absence of drilling fluid where quartz has been considered as the typical formation material. According to the behaviors sketched in this figure, the deviation between the values of *N*_*p*_ for these conditions range between about 29.2 to 33% for the near detector, and between about 37 to 42.9% for the far detector. It can therefore be concluded that for a given thickness of mudcake and formation density, both detectors experience approximately the same percentage of change in counting rate. Also shown in this figure, the values of *N*_*m*_ have been compared with those of the porous model. The deviation between the values of *N*_*m*_ in the presence and absence of drilling fluid range between about 31.4 to 34.7% for the near detector, and between about 47.4 to 50.8% for the far detector. Moreover, the results show that *δ*(*N*_*p*_) is in the range of 5.1 to 8.7%, and within 5.2 to 28%, for near and far detectors, respectively.

## Discussion and Conclusion

Flow and transport phenomena in porous media and fractured rocks, as well as industrial synthetic porous matrices, arise in many diverse fields of science and technology. As an outstanding example, the reservoirs are complex fractured media which the fluid can flow through the porosities. In the petroleum industry, well logs are the main source of information concerning the subsurface formations, providing measurements of the rock formation characteristics and the fluids in the pore spaces. These logging tools often houses a radioisotope as a source that emits radiation that interacts with the surrounding medium. Given that simulation-based studies are very important in designing optimized tools, using the traditional approach for considering the medium surrounding the borehole as a homogeneous mixture of materials for investigation of the effective factors on increasing the yield of the logging tools may be of concern.

Aiming to make clear whether the response of the detectors in the logging tools is affected by the distribution pattern of oil in the medium, the present study dealt with the simulation of a model for porous media. This simplified model includes a large number of matrix-shaped blocks with randomly dispersed oil-filled sites. The results show that for a given formation density, though the count rates corresponding to both mixture and porous model linearly depend on the oil concentration, the parameters relative to the fitted lines are not the same. For quartz as an example, the slope of the straight-lines to the data of porous model deviates from that of the mixture model by 60.8% and 191% for near and far detectors, respectively. However, the intercepts of these lines for two models deviate from each other by about 2.8% and 0.4% for near and far detectors. Owing to the fact that by decrement in the oil concentration, the porous model approximately behaves similar to the traditional mixture model, this result was well expected.

The simulations were extended for different materials as formation. The results show that *δ*(*N*_*p*_) ranges between about −2 to 10% for the near detector, and between about −1 to 22% for the far detector. The results suggest that the response of the far detector is more sensitive to the approach of simulation of the medium in comparison to that of the near detector. Moreover, the more the density of the formation increases, the more the values of *δ*(*N*_*p*_) grow. This increment is more notable for higher oil concentrations.

According to the results, the count rates show a dependency on both oil concentration and the formation material. For all formation materials, the smallest oil concentrations leads to the smaller values of *N*_*p*_. This can be understood as the result of the decrement of the average density of the medium by increment of the oil concentration which causes the facile travel of the gamma rays through the medium.

The dependency of counting rates for various formation densities was investigated to assess the performance of Eq. (1) in the porous model compared with the homogeneous mixture model. The results show that for both models, the natural logarithm of the count rates behaves linearly against the porosity with concentration-dependent slopes. However, owing to that the slopes of these straight-lines are not the same, the formula governing the models are different. According to Eqs. (3) to (6), the slopes of the linear fits corresponding to the porous model deviate from those of the homogeneous mixture model by about 73.3% and 53.8% for near and far detectors, respectively. These deviations for the intercepts are 28.5% and 20%. The mentioned differences are expected to lead the disturbance in the density evaluation.

The effect of the presence of the drilling fluid on the count rate of the porous model was also investigated. According to the results, for a given thickness of mudcake and the formation density, both detectors show approximately the same percentage of change in counting rate. In other words, though the values of *N*_*p*_ are affected by the presence of the drilling fluid, the slopes of their fitted lines almost remain constant. This behavior can also be seen for *N*_*m*_ in the homogeneous mixture model. However, the values of *δ*(*N*_*p*_) show a distinct difference between the performance of two models, especially in far detectors. According to the results presented in Fig. 10, *δ*(*N*_*p*_) is concentration-dependent, varying between 5.1 to 8.7% for near detector. For the farther spacing detector which has more density resolution compared to the shorter spacing detector and consequently its response is more important in density measurement, this quantity ranges from 5.2 to 28%. This result supports the importance of using different models on the well logging simulations.

This work tried to investigate the differences in the behavior of the parameters governing the detector responses in a given logging tool arisen from using different models and to give a prediction of density estimation in the porous media to get closer to the realistic condition. It is worthy to mention that the present results can be extended to simulation of more sophisticated models of the porous media, considering a wider span of formation materials and flowing fluids, as well as different densities and thicknesses of drilling fluids. Also, more work is needed to investigate whether the geometries of the logging tool optimized by using a traditional mixture model is disturbed by employing the porous model. These problems offer new ways to accomplish more researches in simulation-based studies on the well logging technique.
